# A Rare Cause of Transaminitis: Anti-3-Hydroxy-3-Methylglutaryl-CoA Reductase Myopathy

**DOI:** 10.7759/cureus.111300

**Published:** 2026-06-22

**Authors:** Ross M Dies, Peng-Sheng Ting, Munraj Singh

**Affiliations:** 1 Department of Medicine, Gastroenterology Section, Louisiana State University Health Sciences Center, New Orleans, USA; 2 Department of Medicine, Gastroenterology and Hepatology Section, Tulane University School of Medicine, New Orleans, USA

**Keywords:** 3-hydroxy-3-methyl-glutaryl-coenzyme a reductase (anti-hmgcr) antibodies, drug-induced transaminitis, immune-mediated necrotizing myopathy, myotoxicity, statin-induced myopathy

## Abstract

Persistent transaminitis has a very broad differential diagnosis, and extrahepatic causes may often be initially overlooked. Anti-3-hydroxy-3-methylglutaryl-CoA reductase (anti-HMGCR) myopathy is a subtype of immune-mediated necrotizing myopathy (IMNM) characterized by the presence of anti-HMGCR antibodies and myofiber injury. Patients typically present with proximal extremity weakness, transaminitis, and a history of statin use. Unlike statin intolerance or statin myopathy, the weakness and biochemical abnormalities persist despite discontinuation of the drug. Here, we present a case of a patient with transaminitis and elevated creatine kinase (CK) levels, which persisted after cessation of his statin and were found to be associated with mildly elevated anti-HMGCR antibodies. Interestingly, he did not require immunosuppression for the resolution of his myopathy.

## Introduction

Immune-mediated necrotizing myopathy (IMNM) is an autoimmune disease that is associated with specific antibodies, namely, anti-3-hydroxy-3-methylglutaryl-coenzyme A reductase (anti-HMGCR) and anti-signal recognition particle (anti-SRP) antibodies, although some cases are seronegative [1,2]. While anti-SRP-positive IMNM has unclear risk factors, anti-HMGCR IMNM is strongly associated with statin use [1]. Rarely, the disease may occur in statin-naive patients, and the class II HLA allele DRB1*11:01 has been implicated as a genetic risk factor [3]. This immune-mediated process is separate from the direct myotoxic effects associated with statins, from which most patients will recover spontaneously after cessation of the medication [1,4]. Statin-induced IMNM as a condition is usually more severe, and patients will have symmetric bilateral proximal muscle weakness of varying degrees, as well as markedly elevated creatine kinase (CK) levels that will often persist even after the drug is stopped [1,4]. Other laboratory abnormalities include elevated serum transaminases, as these enzymes are also present within muscle cells [5].

Muscle biopsy is not usually required for diagnosis if laboratory values and clinical presentation are compatible; moreover, findings on histopathological specimens can be nonspecific and insensitive [1,6,7]. Newer diagnostic criteria do not require muscle biopsy for diagnosis, and the presence of anti-HMGCR antibodies alone has a specificity approaching 99% [1,6,7]. If obtained, electromyography (EMG) may show small amplitude motor potentials and spontaneous activity characteristic of a myopathic process, and muscle edema may be seen on magnetic resonance imaging (MRI) [3,6]. Recognizing this form of myositis is important, as prompt initiation of immunosuppressive therapy is often needed to halt the disease process and preserve muscle function [7]. Furthermore, there are rare instances where this condition can be self-limiting, with good recovery of muscle function and corresponding improvement in laboratory values without the use of immunosuppression [4,8,9]. Here, we present a case of a patient with anti-HMGCR myopathy that improved after statin cessation and without the use of additional medical therapy.

## Case presentation

A 73-year-old male with a history of hypertension, hyperlipidemia, diabetes mellitus, colon cancer with resection, prostate cancer with radiation therapy on regular leuprolide injections, and hepatic hemangiomas presented to the hepatology clinic as a referral for persistently elevated transaminases. He was first noted to have elevated enzymes eight months prior to our visit, and this was followed in the subsequent weeks by worsening proximal upper and lower-extremity weakness, with difficulty getting in and out of his bathtub and climbing and descending stairs. A CK level at a follow-up visit three months later was markedly elevated. Of note, he had been on atorvastatin 40 mg daily for several years before this without issue, but this medication was promptly discontinued after these findings. Despite statin cessation, his weakness, transaminitis, and CK elevation persisted, although less severely than before (Table 1), while alkaline phosphatase and bilirubin persistently remained within normal limits. In our clinic, he had no jaundice, scleral icterus, or abdominal pain. Physical exam was notable for 4/5 strength on hip flexion by the Medical Research Council scale, as well as 3/5 on trunk flexion. His upper extremities and distal lower extremities demonstrated 5/5 strength. Workup was performed for anti-nuclear, anti-HMGCR, anti-signal recognition particle (SRP), anti-mitochondrial, and anti-smooth muscle antibodies, as well as hepatitis serologies, hemochromatosis analysis, alpha-1-antitrypsin, and abdominal ultrasound. Ultrasound showed coarse hepatic echotexture, hepatopedal flow of the portal vein, and hyperechoic lesions consistent with known hemangiomas. Workup was mostly unremarkable, except for a mildly elevated anti-HMGCR antibody titer. Due to the clinical picture, antibody positivity, and associated statin use, he was presumed to have anti-HMGCR myopathy. With his improving symptoms and laboratory values, along with concern for his diabetes, we did not elect to start him on empiric steroid therapy, nor did we pursue more invasive testing (e.g., muscle biopsy, EMG). Over the next 10 months, his CK levels and transaminases normalized, and his strength returned to his baseline from roughly two years prior. It was recommended that he not be rechallenged with statins, and that consideration be given to initiation of a proprotein/subtilisin/kexin type 9 (PCSK9) inhibitor for lipid management and cardiovascular risk reduction.

**Table 1:** Serial laboratory values demonstrating correlation of resolving transaminitis and creatine kinase Abbreviations: CK, creatine kinase; U/L, units per liter; AST, aspartate aminotransferase; ALT, alanine aminotransferase; anti-HMGCR Ab, anti-3-hydroxy-3-methylglutaryl-coenzyme A reductase antibody; CU, chemiluminescence units; N/A, not available Month 0 represents the date of initial presentation. Statin discontinuation occurred at month 3. Notably, month 8 was the date of the hepatology clinic visit.

Month	CK (U/L)	AST (U/L)	ALT (U/L)	Anti-HMGCR Ab (CU)
0	N/A	89	110	N/A
1	N/A	114	167	N/A
3	11010	202	256	N/A
4	7333	125	169	N/A
8	3751	68	90	25
10	1934	48	71	N/A
12	906	32	45	N/A
15	430	30	42	N/A
18	150	28	37	N/A
Reference range	<230	<45	<46	<20

## Discussion

This case highlights the significance of considering extrahepatic causes of elevated transaminases, as well as differentiating direct statin myotoxicity from antibody-mediated statin myopathy. Here, we report on a patient with anti-HMGCR myopathy that spontaneously resolved with cessation of statin and without the need for immunosuppressive therapy. Most patients require minimal systemic glucocorticoids to achieve clinical stability and prevent relapses, with others requiring the addition of intravenous immunoglobulin (IVIG) and other immunomodulatory agents (e.g., triple therapy) [7,9]. Some data suggest that IVIG may be used as monotherapy in select patients with limited disease and/or diabetes, as well as rescue therapy for those failing initial treatment or with relapse [3,4,6]. Statin discontinuation alone is often not enough to halt the disease process, and timely treatment in those with severe disease is necessary to prevent severe muscle damage and, ultimately, fibrofatty replacement of muscle tissue resulting in permanent weakness [2]. Interestingly, our patient was able to improve without the use of any immunosuppressive medications. Lengthy duration of steroid treatment presents its own challenges, namely, worsening glucose tolerance, which was of concern given our patient’s diabetes. 

Of note, anti-HMGCR myopathy can occur in someone who has been on statin therapy for years, as in this patient [1]. Indeed, a small study by Alvarez-Troncoso et al. demonstrated the mean treatment time before symptom onset at 20.8 (± 23.9) months [10]. CK levels correlate well with disease activity, and monitoring for a steady decline in these levels should be done once a diagnosis is made [7]. Elevated transaminases are often seen, and anti-HMGCR myopathy tends to show alanine transaminase (ALT) predominance and a lower AST:ALT ratio [5]. Muscle biopsy, although not necessary for diagnosis and not readily available in many general hospital settings, typically reveals necrotic fibers and regeneration with associated inflammatory infiltrates, but is inconclusive up to 20% of the time [6,11,12]. EMG is not necessary for diagnosis, cannot differentiate between different myopathies, requires multiple sites for sampling, and creates new sites where muscle biopsy cannot be pursued; it can be useful in excluding neurogenic causes [12]. Antibody titers, while quite useful in determining diagnosis, have less utility in inferring the severity of disease when compared between patients, although they may be useful as a follow-up marker to assess response during immunosuppressive treatment [6,8,13]. Still, these autoantibodies should be obtained, as accurate diagnosis and classification of myositis is helpful in pursuing management and precludes the need for biopsy [13,14]. In addition, the presence of these antibodies is highly specific for anti-HMGCR myopathy, and they are notably absent in statin myotoxicity [3,4].

Although our patient had only mildly elevated titers, his history, recent statin use, clinical picture, and markedly elevated CK levels were convincing evidence of this diagnosis even without muscle biopsy, EMG, or MRI. Identifying patients who may be appropriate for clinical monitoring instead of immunosuppressive treatment is difficult. A recently published retrospective study identified four patients with anti-HMGCR myopathy who spontaneously recovered without immunosuppressive treatment, although these patients all had histopathological specimens to accompany lab findings [15]. The authors were not able to draw any firm conclusions regarding which patients may be appropriate for this management strategy, and they note that spontaneous resolution may not be due to complete remission, but rather a relapsing remitting pattern [15]. Furthermore, relapse during weaning and/or after cessation of immunosuppression is well-documented for anti-HMGCR myopathy, highlighting the potentially chronic nature of the disease and the need for long-term surveillance. Although the exact mechanisms underlying relapse remain poorly defined, environmental triggers such as infection and other immune stressors have been proposed and may contribute to disease activation in susceptible individuals [7,13,15]. CK levels remain a good marker to monitor for relapse [7,9].

In another study by Allenbach et al., three statin-exposed patients with confirmed IMNM improved within months of statin withdrawal without any other intervention, suggesting that some cases of "statin intolerance" may in fact be autoimmune myopathy, and reintroduction of these agents may trigger relapse [16]. MRI may be useful to help monitor such patients and identify subclinical disease or further evaluate those with persistent weakness but normal CK values, with findings of muscle edema, fibro-fatty replacement, and increased short tau inversion recovery (STIR) signals indicating ongoing disease activity [8,13,15]. EMG would not have much value in monitoring these patients, but may be helpful in differentiating a disease flare from possible steroid myopathy in someone receiving those agents for treatment [12]. We are unable at this time to identify exactly why our patient experienced clinical remission without immunosuppression. One contributing factor may be his milder degree of weakness at presentation. Another may be his lower antibody titer, which could possibly indicate milder disease. His anti-HMGCR antibody testing was obtained 5 months after statin discontinuation, which may also imply that his titer had been higher during ongoing administration of the drug and had since downtrended. Regardless, he will require close monitoring for recurrence of symptoms, and he will require strict statin avoidance. Patients with anti-HMGCR myositis should not be re-challenged with an alternative statin, as is done in individuals with statin-associated myalgia, and consideration should be given to starting alternative agents such as ezetimibe, PCSK9 inhibitors, bempedoic acid, or inclisiran for management of dyslipidemia and mitigation of cardiovascular risk [1,10,17,18]. 

In the differential for transaminitis, one must consider sources other than the liver itself [19,20]. This is especially true if other hepatobiliary markers, such as bilirubin, alkaline phosphatase, and gamma-glutamyl transferase, are normal or minimally elevated with markedly elevated CK levels, as this pattern could be more consistent with muscular injury [20]. Consideration should be given to the possibility of statin-associated muscle injury when clinically applicable.

## Conclusions

The recognition of a muscular source of elevated transaminases is vital to avoid misattribution to primary liver disease and to guide appropriate evaluation and management. Persistent transaminitis, markedly elevated creatine kinase, and associated muscle weakness should prompt consideration of anti-HMGCR IMNM, especially in the setting of current or prior statin exposure. This case demonstrates that, although immunosuppressive therapy is often required, select patients with milder presentations and improving symptoms and laboratory markers may have full recovery with statin discontinuation alone and regular monitoring. Management may be individualized based on a patient's clinical trajectory and associated comorbidities. It also should be noted that observation without immunosuppression carries the risk of irreversible muscle damage and should only be considered in carefully selected patients with clear clinical and biochemical improvement. Avoiding future statin therapy and seeking alternative antilipemic agents is essential for both long-term cardiovascular risk reduction and preservation of muscle function.
